# A standard database for drug repositioning

**DOI:** 10.1038/sdata.2017.29

**Published:** 2017-03-14

**Authors:** Adam S. Brown, Chirag J. Patel

**Affiliations:** 1 Department of Biomedical Informatics, Harvard Medical School, 10 Shattuck St, Boston, Massachusetts 02115, USA

**Keywords:** Drug development, Pharmaceutics

## Abstract

Drug repositioning, the process of discovering, validating, and marketing previously approved drugs for new indications, is of growing interest to academia and industry due to reduced time and costs associated with repositioned drugs. Computational methods for repositioning are appealing because they putatively nominate the most promising candidate drugs for a given indication. Comparing the wide array of computational repositioning methods, however, is a challenge due to inconsistencies in method validation in the field. Furthermore, a common simplifying assumption, that all novel predictions are false, is intellectually unsatisfying and hinders reproducibility. We address this assumption by providing a gold standard database, repoDB, that consists of both true positives (approved drugs), and true negatives (failed drugs). We have made the full database and all code used to prepare it publicly available, and have developed a web application that allows users to browse subsets of the data (http://apps.chiragjpgroup.org/repoDB/).

## Background & Summary

Drug repositioning is the process of discovering, validating, and marketing previously approved drugs for new indications. The drug repositioning field is growing rapidly, due to the promise of reduced costs and expedited approval schedules^1^. Unsurprisingly, the number of publications in PubMed with the text ‘drug repositioning’ in their abstracts has ballooned from only 11 articles per year in 2007 to 274 in 2015. Prevalent among repositioning publications are computational methods, which perform *in silico* experiments to determine the most promising repositioning candidates for further preclinical testing^2,3^. Computational repositioning methods have been developed that use a variety of direct and indirect evidence for hypothesis generation, including molecular^4–7^, literature-derived^8–11^, and clinical^12,13^ data. In many computational repositioning methods papers, authors claim that their methods are analytically validated in some way. Authors typically present either case studies, in which they describe a single well-supported example, or sensitivity- and specificity-based analyses to support their claims. Sensitivity- and specificity-based methods rely on comparing the full spectrum of predictions made by a repositioning method to currently approved or investigational drug-indication pairs^14^.

It is difficult, however, to directly compare and/or independently assess computational methods or reported new repositioning candidates due to the variety of analytic validation methodologies favored by different groups (or impossible if only case studies are provided). Furthermore, studies that claim to use unbiased validation methods like predictive ‘area under the receiver-operator curve’ (AUROC)^15^ rely on true, approved drug-indication pairs only, and typically assume that all other drug-indication pairs are false. This assumption is unsatisfying because it relies on a database of approved drugs (the choice of which varies widely in the repositioning literature^14^), and suggests that all novel repositioning predictions are false.

**Figure 1:**
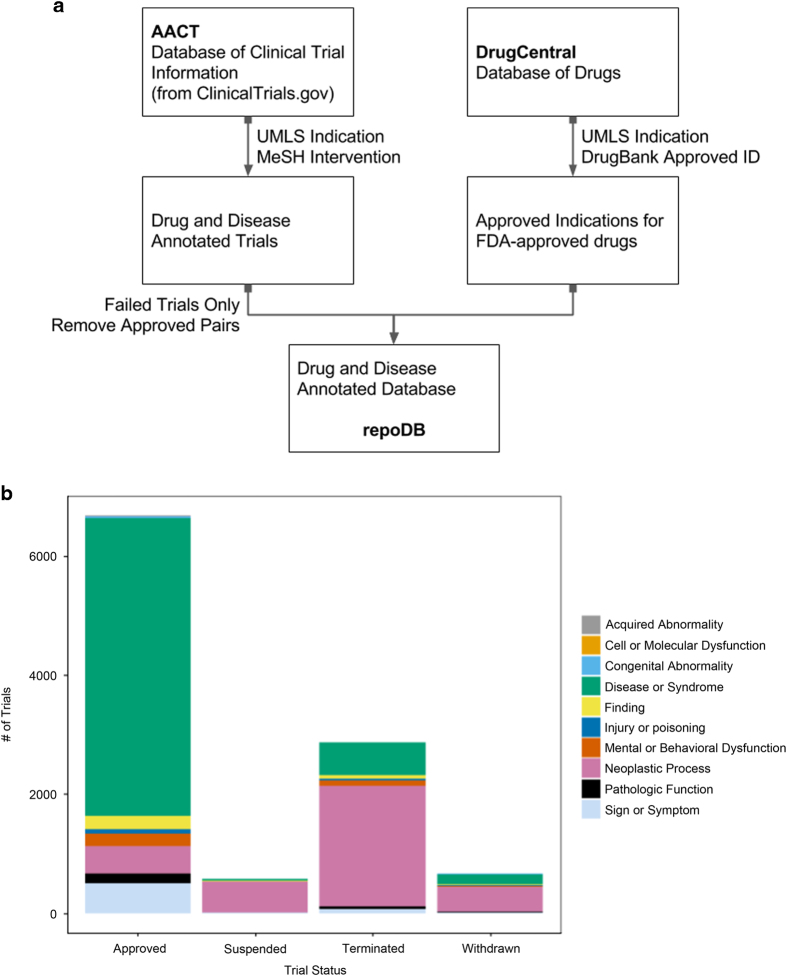
repoDB data sources and database characteristics. (**a**) repoDB data were downloaded from two sources: (1) the AACT indexed version of ClinicalTrials.gov for failed indication information, and (2) DrugCentral for approved indication information. AACT drug-indication pairs were filtered to include only failed pairs, and exclude currently approved pairs. (**b**) The repoDB database contains 6,677 approved drug-indication pairs and 4,123 failed drug-indication pairs. Indications are broken into UMLS semantic types, which describe broad categories of disease. For the two categories with the most records, ‘Disease and Syndrome’ and ‘Neoplastic Process’, we provide lists of the individual terms and their respective numbers in repoDB (see Supplementary Tables 2 and 3).

To address these concerns, we present repoDB, a database of approved and failed drugs and their indications. repoDB approved indications were drawn from DrugCentral, which contains United Medical Language System (UMLS) indications mapped from free-text mentions in drug labels^16,17^. The UMLS is a large biomedical thesaurus that contains information about a wide variety of medical concepts^16^. Failed indications were drawn from the American Association of Clinical Trials Database (the ‘AACT Database’, Clinical Trials Transformation Initiative, 2016), which contains structured records from the National Library of Medicine’s ClinicalTrials.gov database service. Indications in the AACT database are again annotated using medical subject heading (MeSH) terms (a subset of UMLS terms), and represent a mix of investigator-submitted and automatically extracted annotations (see Table 1 for database characteristics and Fig. 1a for an overview of our methodology)^18^. repoDB spans 1,571 drugs and 2,051 UMLS disease concepts, accounting for 6,677 approved and 4,123 failed drug-indication pairs (see Table 2 and Fig. 1b for trial status breakdown). To further assist investigators, we provide a web application (http://apps.chiragjpgroup.org/repoDB/) that enables browsing of the full repoDB database and allows users to download either the full database (or portions relevant to their work). repoDB will enable investigators to not only benchmark their computational repositioning methods, but also gain insight into trends in the drug discovery field and avenues that have not yet been explored.

## Methods

### Approved indication retrieval

As our source of information on currently approved drugs and their indications, we downloaded the full DrugCentral PostgreSQL database, and extracted the tables containing DrugBank identifiers, synonyms for all drugs, and UMLS-mapped indication terms (http://drugcentral.org/, DrugCentral [Full PostgreSQL Database], Data Citation 1)^17^. DrugCentral provides comprehensive information about approved and investigational drugs, including UMLS-mapped approved indication(s) and, important for the construction of repoDB, all synonyms for a given drug. DrugCentral uses the OMOP annotation pipeline to map free text drug labels to UMLS terms, which achieves high annotation accuracy (F1 measures around 0.98)^19^. We retrieved all DrugCentral synonyms for all Food and Drug Administration of the United States (FDA) approved drugs. A list FDA approved drugs was derived from DrugBank, a large drug database that is commonly used by computational drug repositioning methods and is frequently updated with new information (see Fig. 1a)^20^.

### Failed indication retrieval

As our source of information on unsuccessful drug-indication pairs, we downloaded the AACT database from the Clinical Trials Transformation Initiative website (https://www.ctti-clinicaltrials.org/aact-database, March 27, 2016 version, AACT [Pipe Delimited] Data Citation 1)^18^. The AACT database contains structured clinical trial records from the National Library of Medicine’s ClinicalTrials.gov service, and includes information about current trial status and interventions (e.g., drugs, life-style changes) studied in each trial. We chose to use AACT/ClinicalTrials.gov as our source for trial information because the sponsors of most failed trials do not publish their results in the scientific literature (around 78% fail to publish)^21^. We loaded and parsed the full database in R statistical programming environment^22^, and took only those trials that included: 1) an annotated phase between phase 0 and phase 3, 2) a current, overall status of suspended, terminated, or withdrawn, and 3) a MeSH term-mapped intervention (provided by AACT), and 4) a UMLS term-mapped indication (provided by investigators and/or MetaMap analysis of free-text trial descriptions). While the majority of terms are derived from investigator supplied UMLS terms, ClinicalTrials.gov supplements these using the NLM Medical Text Indexer (MTI) to map text to high confidence MeSH/UMLS (F1 measure around 0.55)^23^. We mapped all annotated interventions to DrugCentral synonyms and excluded trials that were not mappable to at least one approved drug (Fig. 1a). Indication information was mapped to UMLS identifiers using the UMLS REST API^16^.

### Final database compilation

**Figure 2:**
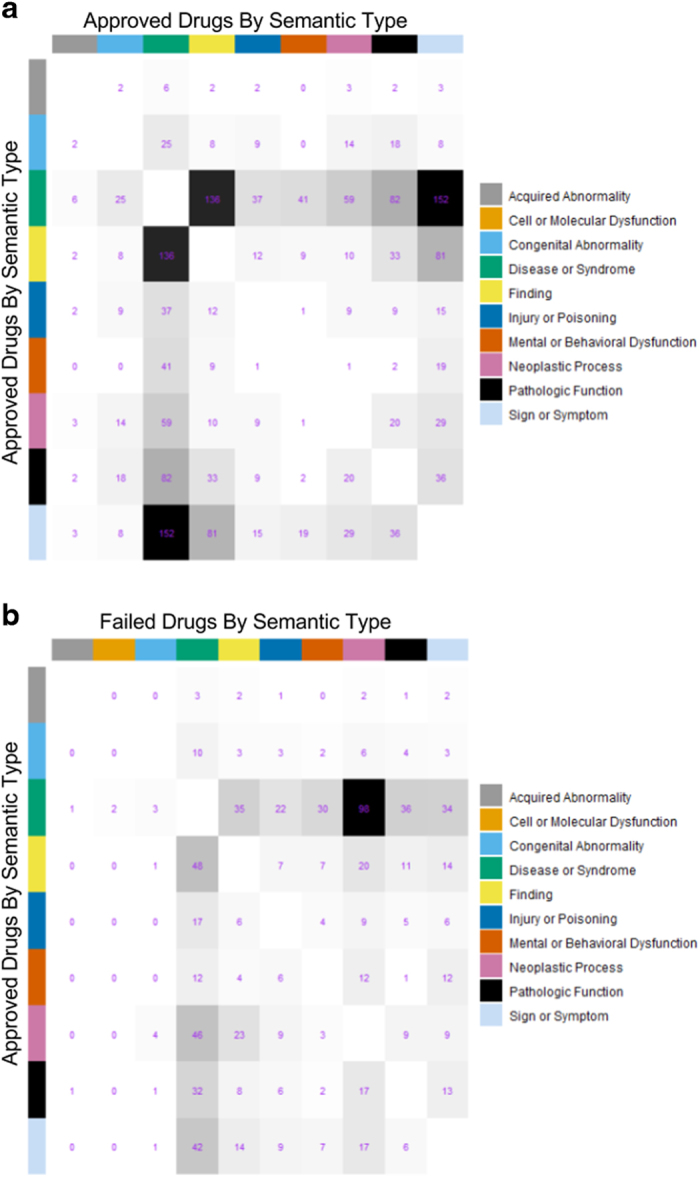
repoDB at-a-glance. Indications were grouped by high-level UMLS ‘Semantic Types,’ which provide insight into broad categories of disease. (**a**) Overlap of approved drugs between semantic types are shown as number of shared drugs. Black indicates the higher overlap and white indicates the lowest (diagonal entries were removed). (**b**) Drugs that are approved for one semantic type and failed in another are shown as above.

As the final step in creating the repoDB database, we reconciled the approved and failed indication information. We removed all failed trial information for drug-indication pairs that were currently approved: for example, metformin is an FDA-approved drug for diabetes mellitus; there are, however, trials marked as terminated with metformin as a primary intervention (e.g., metformin combination therapies, see NCT00762957) and these trials were removed. After combining the approved and failed indications, we kept only those drug-indication pairs for which the indication fell within a UMLS semantic type related to disease ('Disease or Syndrome', 'Neoplastic Process', 'Pathologic Function', 'Finding', 'Mental or Behavioral Dysfunction', 'Sign or Symptom', 'Injury or Poisoning', 'Congenital Abnormality', 'Acquired Abnormality', and 'Cell or Molecular Dysfunction'). Semantic types describe broad categories of disease as well as other medicine-related concepts; it is therefore necessary to filter out non-disease terms, including those with semantic types such as, ‘Health Care Related Organization.’ See Supplementary Table 1 for the highest frequency terms by semantic type. The final database (see Fig. 2) was used to create an interactive *R/Shiny* application (http://apps.chiragjpgroup.org/repodb) whose contents are available for download (Table 2, repoDB [Final Database] Data Citation 1)^24^.

### Code availability

R code used to (1) pre-process DrugCentral and AACT, (2) compile the final repoDB database, and (3) deploy the repoDB R shiny application is available from figshare (repoDB Production Code, Data Citation 1) and from GitHub (https://github.com/adam-sam-brown/repoDB).

## Data Records

We downloaded the full DrugCentral database on November 16, 2016. We have provided a static snapshot of the tables containing DrugBank identifiers, synonyms for all drugs, and UMLS-mapped indication terms used to construct repoDB through figshare in raw form (DrugCentral [Full PostgreSQL Database], Data Citation 1). We downloaded the AACT database of clinical trials on September 2, 2016 (March 27, 2016 version). The database contains structured and free-text fields for 113,571 clinical trials (retrievable using *R*, AACT [Pipe Delimited], Data Citation 1). We have provided a static version through figshare in raw form. The final repoDB database, constructed using the R source code above, spans 1,571 drugs, and 2,051 diseases/indications, accounting for 6,677 approved and 4,123 failed drug-indication pairs. The database is available for download in both comma-separated value and *R* Data format (repoDB [Final Database], Data Citation 1).

## Technical Validation

The drug-indication pairs provided herein are derived from automated annotations of FDA-approved drug labels (in DrugCentral) and investigator-submitted clinical trial records (in AACT). More information about the accuracy of the annotations can be found in the methods, as well as in publications describing the Medical Text Indexer and the Observational Medical Outcomes Partnership (F1 measures of 0.55 and 0.98, used in AACT and DrugCentral respectively)^17–19,23^. We note here that the methods upon which repoDB relies are not the only tools available for named entity recognition in the medical field. Other databases of drug information may therefore vary widely in terms of the granularity (e.g., ‘diabetes’ versus ‘maturity onset diabetes of the young, type I,’ among others) of the indication information contained therein.

## Usage Notes

To ensure that all investigators have easy access to repoDB, we have developed a web application (http://apps.chiragjpgroup.org/repoDB/) using *Shiny* (an *R* Studio project, https://shiny.rstudio.com/). With the *R/Shiny* application, users can:

1. View summary characteristics of repoDB.
2. Search for information about specific drugs, including the indications that have been investigated for that drug, as well as the current status (Approve, Program Terminated, Not Approved, or Trial Halted) and detailed information on the reasons for trial failure (if available). Users can then filter and download results of their search by the current status of the selected drug.
3. Search for information about specific indications, including all drugs that have been investigated for that indication, as well as their current status. As with drug search, users can refine their search by status and download the final search results.
4. Get information on how to cite repoDB.
5. Download the full database.

By using the repoDB database, users agree to cite both our work, as well as both AACT and DrugBank for their role in data curation. This data is available under a Creative Commons Attribution 4.0 International License (see https://creativecommons.org/licenses/by/4.0/ for details).

## Additional Information

**How to cite this article:** Brown, A. S. & Patel, C. J. A standard database for drug repositioning. *Sci. Data* 4:170029 doi: 10.1038/sdata.2017.29 (2017).

**Publisher’s note:** Springer Nature remains neutral with regard to jurisdictional claims in published maps and institutional affiliations.

## Supplementary Material



Supplementary Information

## Figures and Tables

**Table 1:** Characteristics of databases* using in the construction of repoDB

**Source Name**	**Type of Record**	**Number of Records**
DrugCentral	Indication (UMLS)	41,388
AACT	Indication (UMLS)	1,13,571

*Static versions available through figshare (DrugCentral [Full PostgreSQL Database], AACT [Pipe Delimited], Data Citation 1).

**Table 2:** Summary of data available for download through repoDB*

**Indication Status**	**Unique Drugs**	**Unique Diseases**
Approved	1,519	1,229
Terminated	386	785
Withdrawn	199	279
Suspended	77	143

*Also available for download through figshare (repoDB [Final Database], Data Citation 1).
