# Supplementary material for: A standard database for drug repositioning
Source: Sci Data. 2017 Mar 14;4:170029. doi: 10.1038/sdata.2017.29 (PMC5349249; doi:10.1038/sdata.2017.29)
Supplement: Supplementary Information [file sdata201729-s2.docx]

**A Standard Database for Drug Repositioning**

**SUPPLEMENT**

**Table S1.** Number of unique UMLS terms and most common term in repoDB by semantic type

**Table S2.** Breakdown of UMLS terms in the “Disease or Syndrome” UMLS Category

**Table S3.** Breakdown of UMLS terms in the “Neoplastic Process” UMLS Category

**Table S1.** Number of unique UMLS terms and most common term in repoDB by semantic type

| **Semantic Type** | **Number of Unique Terms** | **Most Common Term** | **Number of Term Records** |
| --- | --- | --- | --- |
| Disease or Syndrome | 1079 | Hypertensive disease | 102 |
| Neoplastic Process | 624 | Leukemia | 112 |
| Pathologic Function | 82 | Pulmonary Hypertension | 13 |
| Finding | 74 | Sneezing | 27 |
| Mental or Behavioral Dysfunction | 64 | Schizophrenia | 40 |
| Sign or Symptom | 57 | Pain | 62 |
| Injury or Poisoning | 45 | Corneal Abrasion | 13 |
| Congenital Abnormality | 19 | Diamond-Blackfan Anemia | 10 |
| Acquired Abnormality | 6 | Evidence of liver transplantation | 5 |
| Cell or Molecular Dysfunction | 1 | Oxidative Stress | 2 |

**Table S2**. Breakdown of UMLS terms in the “Disease or Syndrome” UMLS Category

| **UMLS Term** | **Number of Term Records** |
| --- | --- |
| Hypertensive disease | 102 |
| Allergic rhinitis (disorder) | 72 |
| Rheumatoid Arthritis | 64 |
| Infection of skin AND/OR subcutaneous tissue | 63 |
| Allergic Conjunctivitis | 56 |
| Staphylococcus aureus infection | 48 |
| Haemophilus influenzae pneumonia | 45 |
| Urinary tract infection | 44 |
| Common Cold | 37 |
| Diabetes Mellitus, Non-Insulin-Dependent | 37 |
| Escherichia coli urinary tract infection | 37 |
| Pneumonia, Bacterial | 37 |
| Intraabdominal Infections | 36 |
| Lupus Nephritis | 36 |
| Chronic small plaque psoriasis | 34 |
| Seborrheic dermatitis | 34 |
| Streptococcus pyogenes infection | 34 |
| Asthma | 33 |
| HIV Infections | 33 |
| Klebsiella cystitis | 33 |
| Rhinitis, Vasomotor | 33 |
| Bacterial infection due to Klebsiella pneumoniae | 32 |
| Degenerative polyarthritis | 32 |
| Staphylococcal Pneumonia | 32 |
| Bacterial sepsis | 31 |
| Infection of bone | 31 |
| Infective otitis media | 31 |
| Multiple Sclerosis | 31 |
| Epilepsy | 29 |
| Hay fever | 29 |
| Proteus urinary tract infection | 28 |
| Arthropathy associated with infection | 27 |
| Contact Dermatitis | 27 |
| Glaucoma, Open-Angle | 27 |
| Peptic Ulcer | 27 |
| Acute gonococcal cervicitis | 26 |
| Bronchitis | 26 |
| Lower respiratory tract infection | 26 |
| Pneumonia due to Escherichia coli | 26 |
| Acne Vulgaris | 25 |
| Acute gonococcal urethritis | 25 |
| Scalp psoriasis | 25 |
| Granuloma Annulare | 24 |
| Lupus Erythematosus, Discoid | 24 |
| Urticaria | 24 |
| Escherichia coli Infections | 23 |
| Lichen Simplex Chronicus | 23 |
| Psoriasis | 23 |
| Streptococcal pneumonia | 23 |
| Gonorrhea | 22 |
| Pneumonia | 22 |
| Rhinoscleroma | 22 |
| Tinea Pedis | 22 |
| Escherichia coli septicemia | 21 |
| Obesity | 21 |
| Pharyngitis | 21 |
| Tinea corporis (disorder) | 21 |
| Acute exacerbation of chronic bronchitis | 20 |
| Chronic heart failure | 20 |
| Crohn Disease | 20 |
| Gonorrhea of rectum | 20 |
| Lupus Erythematosus, Systemic | 20 |
| Parkinson Disease | 20 |
| Symptomatic dermographism | 20 |
| Acute gonococcal endometritis | 19 |
| Bacterial urinary infection | 19 |
| Enterobacter pneumonia | 19 |
| Sinusitis | 19 |
| Streptococcal tonsillitis | 19 |
| Tinea cruris | 19 |
| Acute otitis media | 18 |
| Ankylosing spondylitis | 18 |
| Bacterial conjunctivitis | 18 |
| Dermatitis, Atopic | 18 |
| Enterobacteriaceae Infections | 18 |
| Gastroesophageal reflux disease | 18 |
| Hepatitis C, Chronic | 18 |
| Ulcerative Colitis | 18 |
| Arthritis, Psoriatic | 17 |
| Headache Disorders | 17 |
| Hypercholesterolemia | 17 |
| Hyperlipidemia | 17 |
| Ocular Hypertension | 17 |
| Osteomyelitis due to Staphylococcus aureus | 17 |
| Female genital tract infection | 16 |
| Migraine Disorders | 16 |
| Moraxella catarrhalis pneumonia | 16 |
| Pneumocystis jiroveci pneumonia | 16 |
| Pneumonia due to Pseudomonas | 16 |
| Mycoplasma pneumonia | 15 |
| Otitis Externa | 15 |
| Pelvic Inflammatory Disease | 15 |
| Peritonitis | 15 |
| Pulmonary Emphysema | 15 |
| Abdominal Abscess | 14 |
| Epilepsy characterized by intractable complex partial seizures | 14 |
| Proteus pneumonia | 14 |
| Rhinitis | 14 |
| Tinea Versicolor | 14 |
| URINARY TRACT INFECTION ENTEROCOCCUS | 14 |
| Blepharoconjunctivitis | 13 |
| Chronic Obstructive Airway Disease | 13 |
| Haemophilus parainfluenzae pneumonia | 13 |
| Tuberculosis, Pulmonary | 13 |
| Acute Coronary Syndrome | 12 |
| Acute Moraxella catarrhalis bronchitis | 12 |
| Arthritis, Gouty | 12 |
| Bacterial keratitis | 12 |
| Bursitis | 12 |
| Chlamydial pneumonia | 12 |
| Hypertriglyceridemia | 12 |
| Hypocalcemia | 12 |
| Infective blepharitis | 12 |
| Inflammatory Bowel Diseases | 12 |
| Osteoporosis | 12 |
| Osteoporosis, Postmenopausal | 12 |
| Serratia Infections | 12 |
| Streptococcal sepsis | 12 |
| Uveitis | 12 |
| Acute bacterial sinusitis | 11 |
| Atrophic Vaginitis | 11 |
| Chancroids | 11 |
| Diabetes Mellitus | 11 |
| Familial hypercholesterolemia - heterozygous | 11 |
| Gastric ulcer | 11 |
| Humoral hypercalcemia of malignancy (disorder) | 11 |
| Lennox-Gastaut syndrome | 11 |
| Parkinsonian Disorders | 11 |
| Pemphigus | 11 |
| Acute tuberculosis | 10 |
| Dermatitis Herpetiformis | 10 |
| Endometriosis | 10 |
| Gonorrhea of pharynx | 10 |
| Herpes zoster keratitis | 10 |
| Hyperlipidemia, Familial Combined | 10 |
| Iridocyclitis | 10 |
| Meningitis, Bacterial | 10 |
| Myocardial Infarction | 10 |
| Nosocomial pneumonia | 10 |
| Pulmonary arterial hypertension | 10 |
| Rosacea | 10 |
| Scurfiness of scalp | 10 |
| Sepsis due to Staphylococcus aureus | 10 |
| Simple Partial Seizures | 10 |
| Syphilis | 10 |
| Tendinitis | 10 |
| Trachoma | 10 |
| Vitamin Deficiency | 10 |
| Absence Epilepsy | 9 |
| Addison Disease | 9 |
| Adrenogenital disorder | 9 |
| Candidiasis, Vulvovaginal | 9 |
| Cutaneous Candidiasis | 9 |
| Cystic Fibrosis | 9 |
| Duodenal Ulcer | 9 |
| Dyslipidemias | 9 |
| Endometritis | 9 |
| Gingivostomatitis | 9 |
| Malaria, Falciparum | 9 |
| Parkinson Disease, Postencephalitic | 9 |
| Sarcoidosis | 9 |
| Sepsis due to Pseudomonas | 9 |
| Staphylococcal bacteraemia | 9 |
| Status Asthmaticus | 9 |
| Synovitis | 9 |
| Yaws | 9 |
| Zollinger-Ellison syndrome | 9 |
| Acquired thrombocytopenia | 8 |
| Actinomycosis | 8 |
| Adrenal cortical hypofunction | 8 |
| Anthrax disease | 8 |
| Asthma attack | 8 |
| Atypical Burkitt's lymphoma | 8 |
| Autoimmune hemolytic anemia | 8 |
| Bladder muscle dysfunction - overactive | 8 |
| Brucellosis | 8 |
| Cerebrovascular accident | 8 |
| Cholangitis | 8 |
| Cooley's anemia | 8 |
| Decompensated cardiac failure | 8 |
| Dermatologic disorders | 8 |
| Erythema Multiforme | 8 |
| Exfoliative dermatitis | 8 |
| Gastroparesis | 8 |
| Granuloma Inguinale | 8 |
| HIV-1 infection | 8 |
| Iritis | 8 |
| Irritable Bowel Syndrome | 8 |
| Keratitis | 8 |
| Legionella pneumophila pneumonia | 8 |
| Meningococcal meningitis | 8 |
| Multiple Sclerosis, Relapsing-Remitting | 8 |
| Musculoskeletal Diseases | 8 |
| Nephrotic Syndrome | 8 |
| Prostatic Hypertrophy | 8 |
| Rheumatic Heart Disease | 8 |
| Serratia sepsis | 8 |
| Serum Sickness | 8 |
| Simple Pulmonary Eosinophilia | 8 |
| Tenosynovitis | 8 |
| Thyroiditis | 8 |
| Tonsillitis | 8 |
| Trichinellosis | 8 |
| Ventricular arrhythmia | 8 |
| Acromegaly | 7 |
| Acute maxillary sinusitis | 7 |
| Addisonian crisis | 7 |
| Aspergillosis | 7 |
| Autoimmune Diseases | 7 |
| Bacterial peritonitis | 7 |
| Candidemia | 7 |
| Candidiasis of the esophagus | 7 |
| Chronic graft-versus-host disease | 7 |
| Chronic idiopathic urticaria | 7 |
| Corneal Ulcer | 7 |
| Cyclitis | 7 |
| Diaper Rash | 7 |
| Disorder of eye | 7 |
| Epicondylitis | 7 |
| Erectile dysfunction | 7 |
| Erosive esophagitis | 7 |
| Familial hypercholesterolemia - homozygous | 7 |
| insufficiency; hepatic, postoperative | 7 |
| Lymphogranuloma Venereum | 7 |
| Narcolepsy | 7 |
| Neonatal meningitis | 7 |
| Neonatal pneumonia | 7 |
| Osteitis Deformans | 7 |
| Proteus septicemia | 7 |
| Psittacosis | 7 |
| Q Fever | 7 |
| Relapsing Fever | 7 |
| Rickettsialpox | 7 |
| Rocky Mountain Spotted Fever | 7 |
| Sepsis of the newborn | 7 |
| Shigella Infections | 7 |
| Status Epilepticus | 7 |
| Streptococcal meningitis | 7 |
| Tonic-Clonic Epilepsy | 7 |
| TYPHUS | 7 |
| Urgency of micturition | 7 |
| URINARY TRACT INFECTION CITROBACTER | 7 |
| Allergic asthma | 6 |
| Arteriosclerosis | 6 |
| Aspiration pneumonitis | 6 |
| Atherosclerosis | 6 |
| Atrial Fibrillation | 6 |
| Atrophy of vulva | 6 |
| Bronchial Spasm | 6 |
| Bronchiectasis | 6 |
| Chlamydia trachomatis infection of genital structure | 6 |
| Chlamydial cervicitis | 6 |
| Chronic Kidney Diseases | 6 |
| Genitourinary tract infection | 6 |
| GONOCOCCAL INFECTION DISSEMINATED | 6 |
| Hemorrhoids | 6 |
| Hepatitis B, Chronic | 6 |
| Herpes zoster disease | 6 |
| Hypertensive urgency | 6 |
| Hyperuricemia | 6 |
| Hypoparathyroidism | 6 |
| Immunologic Deficiency Syndromes | 6 |
| Infection due to Pseudomonas aeruginosa | 6 |
| Interstitial Cystitis | 6 |
| Intrinsic asthma | 6 |
| Iron deficiency anemia | 6 |
| Malaria | 6 |
| Malignant otitis externa due to Pseudomonas aeruginosa | 6 |
| Motion Sickness | 6 |
| Myelofibrosis due to another disorder | 6 |
| Paroxysmal supraventricular tachycardia | 6 |
| Pediculus capitis infestation | 6 |
| Peripheral Vascular Diseases | 6 |
| Persistent Fetal Circulation Syndrome | 6 |
| Primary hypercholesterolemia | 6 |
| Proctitis | 6 |
| prostatitis | 6 |
| Pulmonary Cystic Fibrosis | 6 |
| Respiratory Distress Syndrome, Newborn | 6 |
| Sepsis due to Gram negative bacteria | 6 |
| Staphylococcal meningitis | 6 |
| Systemic candidiasis | 6 |
| Tuberculosis, Miliary | 6 |
| Upper Respiratory Infections | 6 |
| Anemia in chronic kidney disease | 5 |
| Angina Pectoris, Variant | 5 |
| Aphthous Stomatitis | 5 |
| Beckwith-Wiedemann Syndrome | 5 |
| Candidiasis | 5 |
| Cardiac Arrest | 5 |
| Chlamydial urethritis | 5 |
| Clinically isolated syndrome | 5 |
| Cluster Headache | 5 |
| Cytomegalovirus Infections | 5 |
| Deep Vein Thrombosis | 5 |
| Diabetic foot infection | 5 |
| Diarrhoea predominant irritable bowel syndrome | 5 |
| Diffuse Hyperplastic Perilobar Nephroblastomatosis | 5 |
| Extrapyramidal Disorders | 5 |
| Female hypogonadism syndrome | 5 |
| Gaucher Disease | 5 |
| Hb-SS disease with vasoocclusive pain | 5 |
| Hemoglobin SS disease with vasoocclusive crisis | 5 |
| Hypogonadotropic hypogonadism | 5 |
| Infective otitis externa | 5 |
| Inflammatory dermatosis | 5 |
| Inhalational anthrax | 5 |
| Irritable bowel syndrome with diarrhea | 5 |
| Microscopic Polyarteritis | 5 |
| non-gonococcal urethritis (NGU) | 5 |
| Onychomycosis | 5 |
| Oral candidiasis | 5 |
| Other sickle-cell disease with vaso-occlusive pain | 5 |
| Pneumonia due to Klebsiella pneumoniae | 5 |
| Pneumonia due to Staphylococcus aureus | 5 |
| Postherpetic neuralgia | 5 |
| Pulmonary Embolism | 5 |
| Pyelonephritis | 5 |
| Recurrent herpes simplex labialis | 5 |
| Restless Legs Syndrome | 5 |
| Rickets | 5 |
| Staphylococcal endocarditis | 5 |
| Streptococcal endocarditis | 5 |
| Streptococcal Infections | 5 |
| Systemic Scleroderma | 5 |
| Tonic - clonic seizures | 5 |
| Acute bacterial bronchitis | 4 |
| Acute gonococcal epididymo-orchitis | 4 |
| Anterior uveitis | 4 |
| Aplastic Anemia | 4 |
| Aspiration Pneumonia | 4 |
| Bacteremia | 4 |
| Bacterial Infections | 4 |
| Bronchitis, Chronic | 4 |
| candidal peritonitis | 4 |
| Chlamydial pelvic inflammatory disease | 4 |
| Chloasma | 4 |
| Chronic Bacterial Prostatitis | 4 |
| Chronic Fatigue Syndrome | 4 |
| Cystitis | 4 |
| Cystitis escherichia | 4 |
| Dacryocystitis | 4 |
| Deficiency of testosterone biosynthesis | 4 |
| Diabetes Mellitus, Insulin-Dependent | 4 |
| Drug-induced constipation | 4 |
| Dry Eye Syndromes | 4 |
| Endocrine Breast Diseases | 4 |
| Epilepsies, Myoclonic | 4 |
| Epilepsies, Partial | 4 |
| Erysipelas | 4 |
| Exudative age-related macular degeneration | 4 |
| Fibromyalgia | 4 |
| Fusospirochetal pharyngitis | 4 |
| Giardiasis | 4 |
| Gout | 4 |
| Haemophilus sepsis | 4 |
| Hemophilia A | 4 |
| Hepatic Encephalopathy | 4 |
| Herpes Labialis | 4 |
| hormone deficiency | 4 |
| Hyperlipoproteinemia Type III | 4 |
| Hyperthyroidism | 4 |
| Hypogonadism | 4 |
| Hypophosphatemia | 4 |
| Inclusion conjunctivitis | 4 |
| Infective cystitis | 4 |
| Infestation by Phthirus pubis | 4 |
| Influenza | 4 |
| Invasive Pulmonary Aspergillosis | 4 |
| Kidney Failure, Chronic | 4 |
| Listeriosis | 4 |
| Malaria, Vivax | 4 |
| Mastocytosis, Systemic | 4 |
| Meibomianitis | 4 |
| Meningitis | 4 |
| Meningitis, Escherichia coli | 4 |
| Meningitis, Pneumococcal | 4 |
| Mineral deficiency | 4 |
| Onychomycosis of toenails | 4 |
| Ophthalmia Neonatorum | 4 |
| Oropharyngeal candidiasis | 4 |
| Peptic Esophagitis | 4 |
| Periodontitis | 4 |
| Punctate keratitis | 4 |
| Recurrent genital herpes simplex | 4 |
| Renal disease with edema NOS | 4 |
| Scabies <infestation> | 4 |
| Scarlet Fever | 4 |
| Scleroderma | 4 |
| Septicemia due to Bacteroides | 4 |
| Skin Diseases, Infectious | 4 |
| Sleep Initiation and Maintenance Disorders | 4 |
| Tension Headache | 4 |
| Testicular hypogonadism | 4 |
| Traveler's diarrhea | 4 |
| Tuberculosis | 4 |
| Tularemia | 4 |
| Typhoid Fever | 4 |
| Vascular Headaches | 4 |
| Acute Cerebrovascular Accidents | 3 |
| Acute intermittent porphyria | 3 |
| Acute myocardial infarction | 3 |
| Acute osteomyelitis | 3 |
| Adrenal Gland Hyperfunction | 3 |
| African Trypanosomiasis | 3 |
| Alcohol Withdrawal Delirium | 3 |
| Alcohol withdrawal syndrome | 3 |
| Alzheimer's Disease | 3 |
| Amebiasis | 3 |
| Amyloidosis | 3 |
| Anemia, Pernicious | 3 |
| Angioedemas, Hereditary | 3 |
| Angle Closure Glaucoma | 3 |
| Appendicitis | 3 |
| Ascariasis | 3 |
| Asthma-chronic obstructive pulmonary disease overlap syndrome | 3 |
| Atypical Endometrial Hyperplasia | 3 |
| Bacterial Endocarditis | 3 |
| Bacterial Vaginosis | 3 |
| Bartonella Infections | 3 |
| Blastomycosis | 3 |
| Boutonneuse Fever | 3 |
| Brain Diseases | 3 |
| Calcium renal calculus | 3 |
| Cerebral Embolism | 3 |
| Cerebral Ischemia | 3 |
| Cholelithiasis | 3 |
| Cholera | 3 |
| Chronic gouty arthritis | 3 |
| Chronic ulcerative proctitis | 3 |
| Classical phenylketonuria | 3 |
| Clostridium difficile infection | 3 |
| Clostridium Infections | 3 |
| CNS disorder | 3 |
| Coccidioidomycosis | 3 |
| Complicated appendicitis | 3 |
| Condylomata Acuminata | 3 |
| Congestive heart failure | 3 |
| Coronary Artery Disease | 3 |
| Cryopyrin-Associated Periodic Syndromes | 3 |
| Cutaneous anthrax | 3 |
| Cycloplegia | 3 |
| Cytomegalovirus Retinitis | 3 |
| Diabetic Nephropathy | 3 |
| Diabetic peripheral neuropathy | 3 |
| Disorder of rotator cuff | 3 |
| Disorder of shoulder | 3 |
| Disorder of tendon of biceps | 3 |
| Enteric campylobacteriosis | 3 |
| Enterobiasis | 3 |
| Eosinophilic esophagitis | 3 |
| Eosinophilic Pneumonia | 3 |
| Female pelvic cellulitis | 3 |
| Folic Acid Deficiency | 3 |
| Fusariosis | 3 |
| Gastrointestinal anthrax | 3 |
| Giant Cell Arteritis | 3 |
| Gingival Diseases | 3 |
| Graft-vs-Host Disease | 3 |
| Gram-Positive Bacterial Infections | 3 |
| Hamman-Rich syndrome | 3 |
| Hematological Disease | 3 |
| Hemophilia B | 3 |
| Hepatic Coma | 3 |
| Hepatitis C | 3 |
| Histoplasmosis | 3 |
| HSAN Type IV | 3 |
| Hyperammonemia | 3 |
| Hypercalcemia | 3 |
| Hypermagnesemia | 3 |
| Hyperphosphatemia (disorder) | 3 |
| Hypertensive emergency | 3 |
| Hypoalphalipoproteinemias | 3 |
| Hypocalcemic tetany | 3 |
| Impetigo | 3 |
| Infection in solid organ transplant recipients | 3 |
| Juvenile arthritis | 3 |
| Keratitis, Herpetic | 3 |
| Kidney Diseases | 3 |
| Klebsiella sepsis | 3 |
| Klinefelter Syndrome | 3 |
| Latent Tuberculosis | 3 |
| Leishmaniasis, Cutaneous | 3 |
| Liver Cirrhosis | 3 |
| Lung Abscess | 3 |
| Lung diseases | 3 |
| Malignant Carcinoid Syndrome | 3 |
| Malnutrition | 3 |
| Megaloblastic anemia due to folate deficiency | 3 |
| Meniere Disease | 3 |
| Meningitis due to Klebsiella mobilis | 3 |
| Meningitis, Cryptococcal | 3 |
| Myasthenia Gravis | 3 |
| Narcolepsy-Cataplexy Syndrome | 3 |
| Non-Q wave myocardial infarction | 3 |
| Ovarian Diseases | 3 |
| Painful Bladder Syndrome | 3 |
| Paroxysmal atrial fibrillation | 3 |
| pharyngitis due to Haemophilus influenzae | 3 |
| Pleural Effusion, Malignant | 3 |
| Polymyalgia Rheumatica | 3 |
| Postpoliomyelitis Syndrome | 3 |
| Precocious Puberty | 3 |
| Pseudomonas aeruginosa meningitis | 3 |
| Pulmonary Fibrosis | 3 |
| Pulmonary Mycobacterium avium complex infection | 3 |
| Renal Insufficiency | 3 |
| Renal Osteodystrophy | 3 |
| Rickettsia Infections | 3 |
| Secondary glaucoma | 3 |
| Secondary physiologic amenorrhea | 3 |
| Septicemia candida | 3 |
| Septicemia due to enterococcus | 3 |
| Short Bowel Syndrome | 3 |
| Skin Diseases, Bacterial | 3 |
| Soft Tissue Infections | 3 |
| Staphylococcal Infections | 3 |
| Staphylococcal Skin Infections | 3 |
| Stevens-Johnson Syndrome | 3 |
| Tinea Capitis | 3 |
| Toxoplasmosis | 3 |
| Ventricular Fibrillation | 3 |
| Vitamin B 12 Deficiency | 3 |
| Vitiligo | 3 |
| Vulvovaginitis | 3 |
| Acute amebiasis | 2 |
| Acute fungal otitis externa | 2 |
| Allergic otitis externa | 2 |
| Alopecia | 2 |
| Alopecia Areata | 2 |
| Amenorrhea | 2 |
| Amyotrophic Lateral Sclerosis | 2 |
| Anemia of prematurity | 2 |
| Anemia, Megaloblastic | 2 |
| Angiolymphoid hyperplasia | 2 |
| Arthritis, Bacterial | 2 |
| Atrophy of vagina | 2 |
| Bejel | 2 |
| beta Thalassemia | 2 |
| Blood Coagulation Disorders | 2 |
| Body louse infestation | 2 |
| Brill-Zinsser Disease | 2 |
| Cerebrovascular Disorders | 2 |
| Cerebrovascular Occlusion | 2 |
| Chickenpox | 2 |
| Chlamydia Infections | 2 |
| Choroidal retinal neovascularization | 2 |
| Chromoblastomycosis | 2 |
| Chronic idiopathic constipation | 2 |
| Chronic idiopathic neutropenia | 2 |
| Chronic idiopathic thrombocytopenic purpura | 2 |
| Chronic iron overload | 2 |
| Chronic tubotympanic suppurative otitis media | 2 |
| Citrobacter sepsis | 2 |
| Complex dyslipidemia | 2 |
| Conjunctivitis, Vernal | 2 |
| Coronary Arteriosclerosis | 2 |
| Critical Illness | 2 |
| Crohn's disease in remission | 2 |
| CRYPTOCOCCAL INFECTION DISSEMINATED | 2 |
| Cyclic neutropenia | 2 |
| Cystinuria | 2 |
| Diabetic macular edema | 2 |
| Diabetic Neuropathies | 2 |
| Diabetic Retinopathy | 2 |
| Dialysis Catheter Infection | 2 |
| Diverticulitis | 2 |
| dopamine beta hydroxylase deficiency | 2 |
| Empyema, Pleural | 2 |
| Endemic Flea-Borne Typhus | 2 |
| Endomyometritis | 2 |
| Endothelial dysfunction | 2 |
| Enterobacter infection | 2 |
| Enterocolitis, Pseudomembranous | 2 |
| Eosinophilic gastroenteritis | 2 |
| Epileptic drop attack | 2 |
| Erythema nodosum leprosum | 2 |
| Esophageal Diseases | 2 |
| Familial Mediterranean Fever | 2 |
| Fecal Incontinence | 2 |
| Folliculitis | 2 |
| Fungemia | 2 |
| Furunculosis | 2 |
| Gait Disorders, Neurologic | 2 |
| Gas gangrene caused by clostridium perfringens | 2 |
| Gastroenteritis | 2 |
| Gaucher Disease, Type 1 | 2 |
| Genital Herpes | 2 |
| Gilles de la Tourette syndrome | 2 |
| Glaucoma | 2 |
| Gonococcal joint infection | 2 |
| Gonococcal meningitis | 2 |
| Gout acute | 2 |
| Haemophilus influenzae type b infection | 2 |
| Helicobacter-associated gastritis | 2 |
| Heparin-induced thrombocytopenia with thrombosis | 2 |
| Hepatitis B | 2 |
| Hepatitis C recurrent | 2 |
| Hepatolenticular Degeneration | 2 |
| Hyperkeratosis | 2 |
| Hyperprolactinemia | 2 |
| Hyponatremia | 2 |
| Hypoplastic anemia | 2 |
| Hypothyroidism | 2 |
| Infection by Trichuris trichiura | 2 |
| Influenza due to Influenza A virus | 2 |
| invasive aspergillosis | 2 |
| Iron Overload | 2 |
| Irritable bowel syndrome characterized by constipation | 2 |
| Keratitis, Dendritic | 2 |
| Keratoconjunctivitis | 2 |
| Keratoconjunctivitis, Vernal | 2 |
| Klebsiella Infections | 2 |
| Laryngeal Diseases | 2 |
| Leishmaniasis | 2 |
| Leishmaniasis, Visceral | 2 |
| Leprosy | 2 |
| Lichen Planus | 2 |
| Liver Abscess, Amebic | 2 |
| Lupus Erythematosus, Cutaneous | 2 |
| Malaria, antepartum | 2 |
| Malignant essential hypertension | 2 |
| Malignant pericardial effusion | 2 |
| Mucocutaneous candidiasis | 2 |
| Mucormycosis | 2 |
| Myxedema | 2 |
| Neuroaspergillosis | 2 |
| Neurosyphilis | 2 |
| Nocardia Infections | 2 |
| Nonalcoholic Steatohepatitis | 2 |
| Ocular Rosacea | 2 |
| Onchocerciasis | 2 |
| Onychomycosis due to Trichophyton mentagrophytes | 2 |
| Onychomycosis of fingernails | 2 |
| Ophthalmia, Sympathetic | 2 |
| Osteomalacia | 2 |
| Ovale malaria | 2 |
| Overactive Bladder | 2 |
| Paratyphoid Fever | 2 |
| Paronychia Inflammation | 2 |
| Pellagra | 2 |
| Perioperative hypertension | 2 |
| Peripheral Neuropathy | 2 |
| Peyronie Disease | 2 |
| Pinta | 2 |
| Pituitary dwarfism | 2 |
| Plague | 2 |
| Pneumococcal Infections | 2 |
| Pneumococcal pharyngitis | 2 |
| Pneumonia, Viral | 2 |
| Polycystic Kidney Diseases | 2 |
| Polycystic Ovary Syndrome | 2 |
| Postnatal infection | 2 |
| Prediabetes syndrome | 2 |
| Prehypertension | 2 |
| Primary genital syphilis | 2 |
| Pseudomonas aeruginosa infection | 2 |
| Pseudotumor Cerebri | 2 |
| Pure Autonomic Failure | 2 |
| Pyoderma Gangrenosum | 2 |
| Q fever endocarditis | 2 |
| Quartan malaria | 2 |
| Radiculitis | 2 |
| Rat-Bite Fever | 2 |
| Raynaud Disease | 2 |
| Reflex Sympathetic Dystrophy | 2 |
| Respiratory Distress Syndrome, Adult | 2 |
| Respiratory Failure | 2 |
| Respiratory Syncytial Virus Infections | 2 |
| Salmonella sepsis | 2 |
| Scalp Dermatoses | 2 |
| Schistosomiasis | 2 |
| Scrub Typhus | 2 |
| Shoulder tendinitis | 2 |
| Sialorrhea | 2 |
| Skin Ulcer | 2 |
| ST segment elevation myocardial infarction | 2 |
| Streptococcal infection of skin | 2 |
| Strongyloidiasis | 2 |
| Subarachnoid Hemorrhage | 2 |
| Supraventricular tachycardia | 2 |
| Syphilis, Congenital | 2 |
| Syphilis, Latent | 2 |
| Syphilis, secondary | 2 |
| Syphilis, tertiary | 2 |
| Tetanus | 2 |
| Tinea barbae | 2 |
| Toxoplasmosis associated with AIDS | 2 |
| Transformed migraine | 2 |
| Trichomonas Infections | 2 |
| Typhus, Epidemic Louse-Borne | 2 |
| Ulcerative colitis in remission | 2 |
| Urea Cycle Disorders, Inborn | 2 |
| Uric acid renal calculus | 2 |
| Varicosity | 2 |
| Vascular Diseases | 2 |
| Vitamin D-dependent rickets | 2 |
| von Willebrand Disease | 2 |
| Vulvar Vestibulitis | 2 |
| West Syndrome | 2 |
| Xerostomia | 2 |
| 3-Beta-hydroxy-delta-5-C27-steroid dehydrogenase deficiency | 1 |
| Abdominal actinomycosis | 1 |
| Acidosis, Respiratory | 1 |
| Acquired partial lipodystrophy | 1 |
| Acute bacterial epiglottitis | 1 |
| Acute bacterial peritonitis | 1 |
| Acute Disease | 1 |
| Acute epiglottitis | 1 |
| Acute gastroenteritis | 1 |
| Acute GVH disease | 1 |
| Acute otitis externa | 1 |
| Acute pulmonary edema | 1 |
| Acute ST segment elevation myocardial infarction (disorder) | 1 |
| Age related macular degeneration | 1 |
| Allergic keratoconjunctivitis | 1 |
| Allergic urticaria | 1 |
| Alopecia, Male Pattern | 1 |
| Altitude Sickness | 1 |
| Anemia, Sickle Cell | 1 |
| Anterior Ischemic Optic Neuropathy | 1 |
| Anthrax sepsis | 1 |
| Antiphospholipid Syndrome | 1 |
| Apnea in the newborn | 1 |
| Arthritis | 1 |
| Aspergillosis, Allergic Bronchopulmonary | 1 |
| Atrioventricular Block | 1 |
| Bacterial enteritis | 1 |
| Bacterial meningitis due to Gram-negative bacteria | 1 |
| Bacteroides empyema | 1 |
| Biliary Tract Diseases | 1 |
| Biotin deficiency disease | 1 |
| BK virus nephropathy | 1 |
| Brain Abscess | 1 |
| Brain Ischemia | 1 |
| Breast Fibrocystic Disease | 1 |
| Bronchiolitis | 1 |
| Bronchiolitis Obliterans | 1 |
| Bronchopulmonary Dysplasia | 1 |
| Calcinosis cutis | 1 |
| Canavan Disease | 1 |
| Cancer anemia | 1 |
| Candidal endocarditis | 1 |
| Candidal meningitis | 1 |
| Carbamoyl-Phosphate Synthase I Deficiency Disease | 1 |
| Cardiomyopathies | 1 |
| Carotid Artery Diseases | 1 |
| Carotid Stenosis | 1 |
| Carpal Tunnel Syndrome | 1 |
| Cerebral Vasospasm | 1 |
| Cervicofacial actinomycosis | 1 |
| Chemotherapy-induced peripheral neuropathy | 1 |
| Cholecystitis | 1 |
| Chondrodysplasia punctata, X-linked dominant type | 1 |
| Chorioretinitis | 1 |
| Choroiditis | 1 |
| Chronic Infantile Neurological, Cutaneous, and Articular Syndrome | 1 |
| Chronic intestinal amebiasis | 1 |
| Chronic rejection of renal transplant | 1 |
| Chronic thromboembolic pulmonary hypertension | 1 |
| Chronic ulcer of lower extremity | 1 |
| Chronic ulcerative rectosigmoiditis | 1 |
| Chronic urticaria | 1 |
| Churg-Strauss Syndrome | 1 |
| Citrullinemia | 1 |
| Clostridium difficile diarrhea | 1 |
| colorectal disorders | 1 |
| Common wart | 1 |
| Congenital hemolytic uremic syndrome | 1 |
| Conjunctivitis, Giant Papillary | 1 |
| Contact dermatitis due to Rhus diversiloba | 1 |
| Corneal Diseases | 1 |
| Corneal ectasia | 1 |
| Coronary Artery Vasospasm | 1 |
| Cryptosporidiosis | 1 |
| Dapsone resistant mycobacterium leprae disease | 1 |
| Decompensated chronic heart failure | 1 |
| Deficiency of mevalonate kinase | 1 |
| Deglutition Disorders | 1 |
| Delta-4-3-oxosteroid-5-beta-reductase deficiency | 1 |
| Dental caries | 1 |
| Dermatomyositis | 1 |
| Diabetic Angiopathies | 1 |
| Diphtheria | 1 |
| Diphyllobothriasis | 1 |
| Disorder of keratinization | 1 |
| Disorder of vitamin C | 1 |
| Disseminated Intravascular Coagulation | 1 |
| Disseminated sporotrichosis | 1 |
| Diverticulitis of gastrointestinal tract | 1 |
| Drug-induced coagulation inhibitor disorder | 1 |
| Drug-induced mucositis | 1 |
| Dupuytren's disease of finger, with contracture | 1 |
| Dysentery | 1 |
| Dyskeratosis Congenita | 1 |
| Early latent syphilis, positive serology, negative cerebrospinal fluid, less than 2 years after infection | 1 |
| Echinococcus granulosus infection | 1 |
| Echinococcus granulosus infection of liver | 1 |
| Echinococcus granulosus infection of lung | 1 |
| Ecthyma gangrenosum | 1 |
| Eczema | 1 |
| Elephantiasis | 1 |
| Endemic goiter | 1 |
| Endocarditis haemophilus | 1 |
| Enterocolitis | 1 |
| EPILEPSY, PYRIDOXINE-DEPENDENT | 1 |
| Erythrasma | 1 |
| Erythropoietic Protoporphyria | 1 |
| Esotropia | 1 |
| Exacerbation of multiple sclerosis | 1 |
| Exocrine pancreatic insufficiency | 1 |
| Extrinsic allergic alveolitis | 1 |
| Fabry Disease | 1 |
| Factor 8 deficiency, acquired | 1 |
| Factor II deficiency | 1 |
| Familial generalized lipodystrophy | 1 |
| Female stress incontinence | 1 |
| Fibromyalgia, Primary | 1 |
| Filarial Elephantiases | 1 |
| Foot Ulcer | 1 |
| Fungal conjunctivitis | 1 |
| Fungal infection of lung | 1 |
| Fungal keratitis | 1 |
| Fungal septicemia | 1 |
| Gastrointestinal candidiasis | 1 |
| Gastroparesis due to diabetes mellitus | 1 |
| Generalized glycogen storage disease of infants | 1 |
| Generalized Myotonia of Thomsen | 1 |
| Gingivitis | 1 |
| Glaucoma, Primary Open Angle | 1 |
| Glossopharyngeal Neuralgia | 1 |
| Gonococcal endocarditis | 1 |
| Graves Disease | 1 |
| Growth Hormone Secretion Abnormality | 1 |
| Hashimoto Disease | 1 |
| Heart Diseases | 1 |
| Heart Failure, Systolic | 1 |
| Helicobacter pylori infection | 1 |
| Hemoglobin SS disease with crisis | 1 |
| Hemoglobinopathies | 1 |
| Heparin-induced thrombocytopenia | 1 |
| Hepatic Veno-Occlusive Disease | 1 |
| Hepatitis, Alcoholic | 1 |
| Hereditary C1 esterase inhibitor deficiency - deficient factor | 1 |
| Hereditary factor XIII A subunit deficiency | 1 |
| Hereditary hemorrhagic telangiectasia | 1 |
| Hereditary orotic aciduria | 1 |
| Herpes encephalitis | 1 |
| Herpes simplex infection of skin | 1 |
| Herpes Simplex Infections | 1 |
| Herpes simplex keratoconjunctivitis | 1 |
| Herpes zoster keratoconjunctivitis | 1 |
| Hidradenitis Suppurativa | 1 |
| HIV-Associated Lipodystrophy Syndrome | 1 |
| HMN (Hereditary Motor Neuropathy) Proximal Type I | 1 |
| Homocystinuria | 1 |
| Human immunodeficiency virus I infection | 1 |
| Hyperaldosteronism | 1 |
| Hyperinsulinism | 1 |
| Hyperparathyroidism | 1 |
| Hyperparathyroidism, Primary | 1 |
| Hypersomatotropic gigantism | 1 |
| Hypertrichosis of eyelid | 1 |
| Hypertrophic Cardiomyopathy | 1 |
| Hypoglycemia | 1 |
| Hypophosphatasia | 1 |
| Hypopituitarism | 1 |
| Hypoxic respiratory failure | 1 |
| Idiopathic Hypereosinophilic Syndrome | 1 |
| Immune thrombocytopenic purpura | 1 |
| Infant, Small for Gestational Age | 1 |
| Infantile Severe Myoclonic Epilepsy | 1 |
| Infection by Campylobacter fetus | 1 |
| Infection by Cryptococcus neoformans | 1 |
| Infection by Pasteurella multocida | 1 |
| Infection by Uncinaria | 1 |
| Infection due to anaerobic bacteria | 1 |
| Infection due to Erysipelothrix rhusiopathiae (disorder) | 1 |
| Inflammatory disorder | 1 |
| Influenza due to Influenza A virus subtype H1N1 | 1 |
| Intermittent Claudication | 1 |
| Intracranial Arteriosclerosis | 1 |
| Invasive Fungal Infections | 1 |
| Iron Metabolism Disorders | 1 |
| Ischemic priapism | 1 |
| Ischemic stroke | 1 |
| Jaundice, Obstructive | 1 |
| Juvenile Spinal Muscular Atrophy | 1 |
| Keratoconjunctivitis Sicca | 1 |
| Kidney Failure | 1 |
| Lactose Intolerance | 1 |
| Lambert-Eaton Myasthenic Syndrome | 1 |
| Laron Syndrome | 1 |
| Larva Migrans, Cutaneous | 1 |
| Late latent syphilis | 1 |
| Latent yaws | 1 |
| Legionnaires' Disease | 1 |
| Leprosy, Multibacillary | 1 |
| Letterer-Siwe Disease | 1 |
| Leukoencephalopathy, Progressive Multifocal | 1 |
| Leukomalacia, Periventricular | 1 |
| Lipid Metabolism, Inborn Errors | 1 |
| Lipodystrophy due to Human immunodeficiency virus infection and antiretroviral therapy | 1 |
| Listerial endocarditis | 1 |
| Liver Abscess | 1 |
| Loiasis | 1 |
| Lyme Disease | 1 |
| Malignant glaucoma | 1 |
| Malignant hyperpyrexia due to anesthesia | 1 |
| Meibomian gland dysfunction | 1 |
| Membranous glomerulonephritis | 1 |
| Meningitis due to Bacteroides | 1 |
| Meningitis, Fungal | 1 |
| Meningitis, Listeria | 1 |
| Meningococcemia | 1 |
| Metabolic Diseases | 1 |
| Metabolic Syndrome X | 1 |
| Missed abortion | 1 |
| mixed; epileptic | 1 |
| Morphea | 1 |
| Motor cortex epilepsy | 1 |
| MRSA - Methicillin resistant Staphylococcus aureus infection | 1 |
| Mucocutaneous leishmaniasis | 1 |
| Mucopolysaccharidosis I | 1 |
| Mucopolysaccharidosis II | 1 |
| Mucopolysaccharidosis VI | 1 |
| Mucopolysaccharidosis, MPS-IV-A | 1 |
| Muscular Atrophy, Spinal, Type II | 1 |
| Muscular Dystrophy, Duchenne | 1 |
| Myasthenia Gravis, Ocular | 1 |
| Mycetoma | 1 |
| Mycotic endocarditis | 1 |
| Myxedema coma | 1 |
| Nasal Polyps | 1 |
| Necrobiosis Lipoidica Diabeticorum | 1 |
| Necrotizing Ulcerative Gingivitis | 1 |
| Neonatal Abstinence Syndrome | 1 |
| Neonatal hypocalcemia | 1 |
| Nephropathic cystinosis | 1 |
| Nesidioblastosis | 1 |
| Neurocysticercosis | 1 |
| Neurogenic Urinary Bladder | 1 |
| Neuropathic diabetic ulcer - foot | 1 |
| Niemann-Pick Disease, Type C | 1 |
| Nocturia | 1 |
| Non-alcoholic Fatty Liver Disease | 1 |
| Non-infective diarrhea | 1 |
| Non-ischemic cardiomyopathy | 1 |
| Noonan Syndrome | 1 |
| Nummular eczema | 1 |
| Nutritional deficiency associated with AIDS | 1 |
| Ocular histoplasmosis syndrome | 1 |
| Onychomycosis due to Trichophyton rubrum | 1 |
| Opisthorchiasis | 1 |
| Optic Atrophy, Hereditary, Leber | 1 |
| Optic Neuropathy, Ischemic | 1 |
| Osteoarthritis, Knee | 1 |
| Osteomyelitis | 1 |
| Other acute postoperative pain | 1 |
| Pancreatic Cyst | 1 |
| Paracoccidioidomycosis | 1 |
| Paralytic Ileus | 1 |
| Paroxysmal hypertension | 1 |
| Paroxysmal nocturnal hemoglobinuria | 1 |
| Pasteurella Infections | 1 |
| Pelvic abscess | 1 |
| Pelvic inflammatory disease due to Mycoplasma hominis | 1 |
| Pemphigus Vulgaris | 1 |
| Peptostreptococcus infection | 1 |
| Perianal warts | 1 |
| Persistent pulmonary hypertension | 1 |
| Pertussis | 1 |
| Phantom Limb Syndrome | 1 |
| Pituitary-dependent Cushing's disease | 1 |
| Plague, Septicemic | 1 |
| Plantar wart | 1 |
| Plasmodium falciparum infection | 1 |
| Pneumonia due to Gram negative bacteria | 1 |
| Pneumonia, Necrotizing | 1 |
| Pneumonia, Ventilator-Associated | 1 |
| Pneumonic Plague | 1 |
| Polyneuropathy | 1 |
| Polyradiculoneuropathy, Chronic Inflammatory Demyelinating | 1 |
| Post-surgical hypoparathyroidism | 1 |
| Posterior synechiae | 1 |
| Postoperative ileus | 1 |
| Pregnancy Complications | 1 |
| Premature Obstetric Labor | 1 |
| Priapism | 1 |
| Primary biliary cirrhosis | 1 |
| primary disorders | 1 |
| Primary fibromyalgia syndrome | 1 |
| Primary hypogonadism | 1 |
| progressive keratoconus | 1 |
| Progressive supranuclear palsy | 1 |
| Proliferative diabetic retinopathy | 1 |
| Proteus meningitis | 1 |
| Pulmonary actinomycosis | 1 |
| Pulmonary cryptococcosis | 1 |
| Pulmonary Edema | 1 |
| Pulmonary Thromboembolisms | 1 |
| Pure Red-Cell Aplasia | 1 |
| Purpura, Thrombotic Thrombocytopenic | 1 |
| Recurrent disease | 1 |
| Renal tubular acidosis | 1 |
| Respiratory Tract Infections | 1 |
| Retinal Diseases | 1 |
| Retinitis Pigmentosa | 1 |
| Rheumatism | 1 |
| Rhinitis, Allergic, Perennial | 1 |
| Rhinocerebral phycomycosis | 1 |
| Rhinosinusitis | 1 |
| Right-sided Staphylococcus aureus endocarditis | 1 |
| Salmonella infections | 1 |
| Salpingitis | 1 |
| Sarcopenia | 1 |
| Senile lentigo | 1 |
| Sensorineural Hearing Loss (disorder) | 1 |
| Septic arthritis haemophilus | 1 |
| Septicemia due to anaerobes | 1 |
| Severe chronic obstructive pulmonary disease | 1 |
| Severe persistent asthma | 1 |
| Sexually Transmitted Diseases | 1 |
| shoulder bursitis | 1 |
| Sjogren's Syndrome | 1 |
| Sleep Apnea, Obstructive | 1 |
| Smith-Lemli-Opitz Syndrome | 1 |
| Staphylococcal enterocolitis | 1 |
| Staphylococcal pharyngitis | 1 |
| Staphylococcal tonsillitis | 1 |
| Stomatitis | 1 |
| Streptococcal toxic shock syndrome | 1 |
| Subacute bursitis | 1 |
| Sucrase-isomaltase deficiency, congenital | 1 |
| Superficial bacterial infection of skin | 1 |
| Systemic onset juvenile chronic arthritis | 1 |
| Teniasis | 1 |
| Tertiary lesion of yaws | 1 |
| Tetany | 1 |
| Thalassemia | 1 |
| Thiamine Deficiency | 1 |
| Thrombasthenia | 1 |
| Thrombocythemia, Essential | 1 |
| Thrombocytopenia | 1 |
| Thyroid Crisis | 1 |
| Tinea | 1 |
| Tinea manus | 1 |
| Toxic Epidermal Necrolysis | 1 |
| Toxocariasis | 1 |
| Toxoplasmosis, Congenital | 1 |
| Transfusion hemosiderosis | 1 |
| Trigeminal Neuralgia | 1 |
| Tuberculosis, Drug-Resistant | 1 |
| Tuberculosis, Meningeal | 1 |
| Tubo-ovarian abscess | 1 |
| Turner Syndrome | 1 |
| Type 2 diabetes mellitus with established diabetic nephropathy | 1 |
| Tyrosinemia, Type I | 1 |
| Urethral atrophy | 1 |
| Urethritis | 1 |
| Urinary Bladder Diseases | 1 |
| Urinary tract infection fungal | 1 |
| Urologic Diseases | 1 |
| Uterine Cervicitis | 1 |
| Vaginal ulcer | 1 |
| Varicose Ulcer | 1 |
| Vascular inflammations | 1 |
| viral; infection, enterovirus | 1 |
| Vitamin A Deficiency | 1 |
| Vitamin B 6 Deficiency | 1 |
| Vitamin D-Resistant Rickets, X-Linked | 1 |
| Vitamin D Deficiency | 1 |
| Vitamin E Deficiency | 1 |
| Vitreomacular traction syndrome | 1 |
| Von Hippel-Lindau Syndrome | 1 |
| Vulvodynia | 1 |
| Wolman Disease | 1 |
| Xanthomatosis, Cerebrotendinous | 1 |
| Zinc deficiency | 1 |

**Table S3.** Breakdown of UMLS terms in the “Neoplastic Process” UMLS Category

| **UMLS Term** | **Number of Term Records** |
| --- | --- |
| leukemia | 112 |
| Malignant neoplasm of lung | 82 |
| Malignant tumor of colon | 69 |
| Multiple Myeloma | 67 |
| unspecified adult solid tumor, protocol specific | 43 |
| Non-Small Cell Lung Carcinoma | 40 |
| Lymphoma, Non-Hodgkin | 35 |
| Lymphoma, T-Cell, Cutaneous | 31 |
| Recurrent Rectal Cancer | 31 |
| Hodgkin Disease | 29 |
| Neuroblastoma | 29 |
| adult acute myeloid leukemia with 11q23 (MLL) abnormalities | 27 |
| Colon cancer recurrent | 26 |
| adult acute myeloid leukemia with inv(16)(p13;q22) | 24 |
| adult acute myeloid leukemia with t(16;16)(p13;q22) | 24 |
| adult acute myeloid leukemia with t(8;21)(q22;q22) | 24 |
| Leukemia, Myelocytic, Acute | 24 |
| Malignant neoplasm of stomach | 24 |
| Non-small cell lung cancer metastatic | 24 |
| recurrent adult acute myeloid leukemia | 24 |
| Non-small cell lung cancer recurrent | 23 |
| Solid tumour | 23 |
| B-cell small lymphocytic lymphoma recurrent | 22 |
| secondary acute myeloid leukemia | 22 |
| Secondary malignant neoplasm of liver | 22 |
| ovarian neoplasm | 21 |
| Stage IV Pancreatic Cancer | 20 |
| Acute lymphocytic leukemia | 19 |
| Colorectal Neoplasms | 19 |
| Monocytoid B-cell lymphoma | 19 |
| Mucosa-Associated Lymphoid Tissue Lymphoma | 19 |
| recurrent grade 1 follicular lymphoma | 19 |
| recurrent grade 2 follicular lymphoma | 19 |
| Pancreatic carcinoma stage III | 18 |
| Splenic Marginal Zone B-Cell Lymphoma | 18 |
| Chronic lymphocytic leukaemia refractory | 17 |
| Glioblastoma | 17 |
| Adenocarcinoma | 16 |
| recurrent adult diffuse large cell lymphoma | 16 |
| recurrent grade 3 follicular lymphoma | 16 |
| Malignant neoplasm of ovary | 15 |
| Marginal zone lymphoma recurrent | 15 |
| Metastatic melanoma | 15 |
| Neoplasm Metastasis | 15 |
| adult acute myeloid leukemia in remission | 14 |
| adult acute myeloid leukemia with t(15;17)(q22;q12) | 14 |
| Colorectal cancer metastatic | 14 |
| Lymphoma, Follicular | 14 |
| Malignant lymphoma of testis | 14 |
| Mantle cell lymphoma recurrent | 14 |
| recurrent adult Burkitt's lymphoma | 14 |
| adult acute megakaryoblastic leukemia (M7) | 13 |
| adult acute monoblastic leukemia (M5a) | 13 |
| adult acute monocytic leukemia (M5b) | 13 |
| adult acute myeloblastic leukemia with maturation (M2) | 13 |
| Adult Acute Myeloid Leukemia without Maturation | 13 |
| adult acute myelomonocytic leukemia (M4) | 13 |
| adult erythroleukemia (M6a) | 13 |
| adult pure erythroid leukemia (M6b) | 13 |
| Angioimmunoblastic Lymphadenopathy | 13 |
| contiguous stage II adult diffuse large cell lymphoma | 13 |
| Cutaneous T-cell lymphoma recurrent | 13 |
| Ki-1+ Anaplastic Large Cell Lymphoma | 13 |
| Melanoma recurrent | 13 |
| mucinous adenocarcinoma of rectum | 13 |
| Mycosis Fungoides | 13 |
| noncontiguous stage II adult diffuse large cell lymphoma | 13 |
| Peripheral T-Cell Lymphoma | 13 |
| Renal Cell Carcinoma | 13 |
| signet ring adenocarcinoma of the rectum | 13 |
| Stage IVA Rectal Cancer | 13 |
| Stage IVB Rectal Cancer | 13 |
| adult acute minimally differentiated myeloid leukemia (M0) | 12 |
| Adult T-cell lymphoma/leukaemia recurrent | 12 |
| chronic myelogenous leukemia in relapse | 12 |
| Mycosis fungoides/Sezary syndrome recurrent | 12 |
| Non-small cell lung cancer stage IIIA | 12 |
| recurrent adult diffuse mixed cell lymphoma | 12 |
| recurrent adult diffuse small cleaved cell lymphoma | 12 |
| recurrent adult immunoblastic large cell lymphoma | 12 |
| recurrent adult lymphoblastic lymphoma | 12 |
| Squamous cell carcinoma | 12 |
| unspecified childhood solid tumor, protocol specific | 12 |
| Acute Myeloid Leukemia Arising from Previous Myelodysplastic Syndrome | 11 |
| Adenocarcinoma of colon | 11 |
| Adenocarcinoma of rectum | 11 |
| Carcinoma breast stage IV | 11 |
| Chronic Lymphocytic Leukemia | 11 |
| Chronic myeloproliferative disorder | 11 |
| Esophageal Neoplasms | 11 |
| Hairy cell leukaemia recurrent | 11 |
| Malignant lymphoma, lymphocytic, intermediate differentiation, diffuse | 11 |
| recurrent adult acute lymphoblastic leukemia | 11 |
| recurrent adult grade III lymphomatoid granulomatosis | 11 |
| recurrent adult Hodgkin's lymphoma | 11 |
| stage IV adult diffuse large cell lymphoma | 11 |
| T-Cell Large Granular Lymphocyte Leukemia | 11 |
| adult nasal type extranodal NK/T-cell lymphoma | 10 |
| Bladder cancer stage III | 10 |
| Breast cancer recurrent | 10 |
| Cutaneous B-Cell Non-Hodgkin Lymphoma | 10 |
| de novo myelodysplastic syndromes | 10 |
| Ewings sarcoma | 10 |
| Hepatosplenic T-cell lymphoma | 10 |
| previously treated myelodysplastic syndromes | 10 |
| Small cell carcinoma of lung | 10 |
| Small Intestinal Lymphoma | 10 |
| stage III adult diffuse large cell lymphoma | 10 |
| Advanced cancer | 9 |
| Breast cancer stage II | 9 |
| Carcinoma | 9 |
| Malignant epithelial neoplasm of female breast | 9 |
| Pancreatic adenocarcinoma metastatic | 9 |
| adult acute lymphoblastic leukemia in remission | 8 |
| Hormone receptor positive malignant neoplasm of breast | 8 |
| Malignant melanoma, metastatic | 8 |
| Mucinous adenocarcinoma of colon | 8 |
| Myelodysplastic/myeloproliferative neoplasm, unclassifiable | 8 |
| Myeloproliferative disease | 8 |
| secondary myelodysplastic syndromes | 8 |
| signet ring adenocarcinoma of the colon | 8 |
| stage I adult diffuse large cell lymphoma | 8 |
| Stage IVA Colon Cancer | 8 |
| Stage IVB Colon Cancer | 8 |
| Acute Promyelocytic Leukemia | 7 |
| Adenocarcinoma Of Esophagus | 7 |
| Adenocarcinoma of prostate | 7 |
| adult acute basophilic leukemia | 7 |
| adult acute eosinophilic leukemia | 7 |
| Colorectal Carcinoma | 7 |
| Kidney Neoplasm | 7 |
| Malignant Glioma | 7 |
| melanoma | 7 |
| Oropharyngeal squamous cell carcinoma stage III | 7 |
| Post-transplant lymphoproliferative disorder | 7 |
| Recurrent Primary Peritoneal Carcinoma | 7 |
| Regional Urothelial Carcinoma of the Renal Pelvis and Ureter | 7 |
| Actinic keratosis | 6 |
| Anaplastic thyroid carcinoma | 6 |
| B-Cell Lymphomas | 6 |
| B-cell small lymphocytic lymphoma stage IV | 6 |
| Benign Prostatic Hyperplasia | 6 |
| Breast Carcinoma | 6 |
| Central Nervous System Neoplasms | 6 |
| Chronic lymphocytic leukaemia stage 3 | 6 |
| Chronic lymphocytic leukaemia stage 4 | 6 |
| Cutaneous Melanoma | 6 |
| Diffuse Large B-Cell Lymphoma | 6 |
| duct cell adenocarcinoma of the pancreas | 6 |
| Epithelial ovarian cancer | 6 |
| Fallopian Tube Neoplasms | 6 |
| Inflammatory Breast Carcinoma | 6 |
| Laryngeal squamous cell carcinoma recurrent | 6 |
| Laryngeal squamous cell carcinoma stage III | 6 |
| Laryngeal verrucous carcinoma recurrent | 6 |
| Liver neoplasms | 6 |
| Malignant neoplasm of prostate | 6 |
| Metastasis from malignant tumor of colon | 6 |
| Myeloid Leukemia, Chronic | 6 |
| Oropharyngeal squamous cell carcinoma recurrent | 6 |
| Ovarian Serous Adenocarcinoma | 6 |
| Papillary serous cystadenocarcinoma | 6 |
| Primary Peritoneal Serous Adenocarcinoma | 6 |
| Prolymphocytic Leukemia | 6 |
| recurrent squamous cell carcinoma of the lip and oral cavity | 6 |
| recurrent squamous cell carcinoma of the paranasal sinus and nasal cavity | 6 |
| Squamous cell carcinoma of the hypopharynx recurrent | 6 |
| Squamous cell carcinoma of the hypopharynx stage III | 6 |
| Squamous cell carcinoma of the hypopharynx stage IV | 6 |
| stage III squamous cell carcinoma of the lip and oral cavity | 6 |
| Testicular Germ Cell Tumor | 6 |
| Verrucous carcinoma of the oral cavity recurrent | 6 |
| Adenocarcinoma of the gastroesophageal junction | 5 |
| adult giant cell glioblastoma | 5 |
| adult glioblastoma | 5 |
| adult gliosarcoma | 5 |
| Adult Kidney Wilms Tumor | 5 |
| anterior urethral cancer | 5 |
| B-cell small lymphocytic lymphoma stage III | 5 |
| Bladder Neoplasm | 5 |
| Childhood Kidney Wilms Tumor | 5 |
| gliosarcoma | 5 |
| Laryngeal squamous cell carcinoma stage IV | 5 |
| malignant melanoma of skin stage III | 5 |
| Malignant neoplasm of ureter | 5 |
| Metastatic Prostate Carcinoma | 5 |
| Multiple Endocrine Neoplasia | 5 |
| posterior urethral cancer | 5 |
| Prostate cancer recurrent | 5 |
| recurrent adult soft tissue sarcoma | 5 |
| recurrent childhood acute lymphoblastic leukemia | 5 |
| recurrent metastatic squamous neck cancer with occult primary | 5 |
| Recurrent Salivary Gland Cancer | 5 |
| Recurrent Urethral Cancer | 5 |
| Secondary malignant neoplasm of lung | 5 |
| squamous cell carcinoma of salivary gland | 5 |
| stage I Wilms' tumor | 5 |
| stage II Wilms' tumor | 5 |
| Stage IIA Rectal Cancer | 5 |
| Stage IIB Rectal Cancer | 5 |
| Stage IIC Rectal Cancer | 5 |
| stage III adult soft tissue sarcoma | 5 |
| stage III Wilms' tumor | 5 |
| Stage IIIA Rectal Cancer | 5 |
| Stage IIIB Rectal Cancer | 5 |
| Stage IIIC Rectal Cancer | 5 |
| stage IV squamous cell carcinoma of the lip and oral cavity | 5 |
| stage IV squamous cell carcinoma of the oropharynx | 5 |
| stage IV Wilms' tumor | 5 |
| stage V Wilms' tumor | 5 |
| Transitional cell cancer of the renal pelvis and ureter localised | 5 |
| untreated childhood supratentorial primitive neuroectodermal tumors | 5 |
| urethral carcinoma associated with invasive bladder cancer | 5 |
| Adenocarcinoma of pancreas | 4 |
| Adult Acute Myeloid Leukemia with inv(16)(p13.1q22); CBFB-MYH11 | 4 |
| Adult Acute Myeloid Leukemia with t(8;21)(q22;q22); RUNX1-RUNX1T1 | 4 |
| adult anaplastic astrocytoma | 4 |
| adult anaplastic oligodendroglioma | 4 |
| Adult Erythroleukemia | 4 |
| Adult Pure Erythroid Leukemia | 4 |
| Alkylating Agent-Related Acute Myeloid Leukemia | 4 |
| Anaplastic astrocytoma | 4 |
| Astrocytoma | 4 |
| Burkitt Lymphoma | 4 |
| Cancer of Head and Neck | 4 |
| Carcinoma of unknown primary | 4 |
| Conventional (Clear Cell) Renal Cell Carcinoma | 4 |
| Endometrial Carcinoma | 4 |
| extragonadal germ cell tumor | 4 |
| HER-2 positive breast cancer | 4 |
| Kaposi Sarcoma | 4 |
| Leukemia, Lymphocytic, Acute, L1 | 4 |
| Leukemia, Myeloid, Accelerated Phase | 4 |
| Nasopharyngeal squamous cell carcinoma stage III | 4 |
| Nasopharyngeal squamous cell carcinoma stage IV | 4 |
| Neuroectodermal Tumor, Primitive | 4 |
| Non-small cell lung cancer stage IIIB | 4 |
| Oestrogen receptor positive breast cancer | 4 |
| Ovarian epithelial cancer recurrent | 4 |
| Pancreatic carcinoma recurrent | 4 |
| Philadelphia chromosome positive chronic myelogenous leukemia | 4 |
| recurrent adult brain tumor | 4 |
| recurrent ovarian germ cell tumor | 4 |
| Recurrent Thyroid Carcinoma | 4 |
| Retinoblastoma | 4 |
| Stage IIB Non-Small Cell Lung Carcinoma | 4 |
| stage III squamous cell carcinoma of the paranasal sinus and nasal cavity | 4 |
| Stage IIIC Skin Melanoma | 4 |
| stage IV adult Burkitt's lymphoma | 4 |
| T-cell lymphoma recurrent | 4 |
| T-cell lymphoma refractory | 4 |
| Waldenstrom Macroglobulinemia | 4 |
| Acute leukemia in remission | 3 |
| Adenocarcinoma of lung (disorder) | 3 |
| advanced adult primary liver cancer | 3 |
| Biliary Tract Cancer | 3 |
| Blast Phase | 3 |
| central nervous system tumor, pediatric | 3 |
| childhood acute basophilic leukemia | 3 |
| childhood acute eosinophilic leukemia | 3 |
| childhood acute erythroleukemia (M6) | 3 |
| childhood acute megakaryocytic leukemia (M7) | 3 |
| childhood acute myeloblastic leukemia with maturation (M2) | 3 |
| childhood acute myeloblastic leukemia without maturation (M1) | 3 |
| childhood acute myelomonocytic leukemia (M4) | 3 |
| Chronic lymphocytic leukaemia stage 1 | 3 |
| Chronic lymphocytic leukaemia stage 2 | 3 |
| Cutaneous T-cell lymphoma stage IV | 3 |
| Endometrial adenocarcinoma | 3 |
| Endometrial cancer stage II | 3 |
| Epidermal growth factor receptor positive non-small cell lung cancer | 3 |
| Esophageal neoplasm metastatic | 3 |
| Gastric carcinoma stage IV | 3 |
| Gastrointestinal Stromal Tumors | 3 |
| Gestational Trophoblastic Neoplasms | 3 |
| HER2-positive carcinoma of breast | 3 |
| Hormone refractory prostate cancer | 3 |
| Intracranial Meningioma | 3 |
| Large cell carcinoma of lung | 3 |
| Laryngeal verrucous carcinoma stage III | 3 |
| leiomyosarcoma | 3 |
| Leukemia, Myeloid, Chronic-Phase | 3 |
| localized unresectable adult primary liver cancer | 3 |
| Lymphoproliferative Disorders | 3 |
| Malignant neoplasm of stomach stage IV | 3 |
| Malignant neoplasm of thyroid | 3 |
| Malignant neoplasm of urinary bladder | 3 |
| Metastatic Renal Cell Cancer | 3 |
| Mixed Oligodendroglioma-Astrocytoma | 3 |
| multiple myeloma in relapse | 3 |
| Myeloid Leukemia | 3 |
| Nasopharyngeal squamous cell carcinoma recurrent | 3 |
| Neoplasm, Residual | 3 |
| Nephroblastoma | 3 |
| Neuroendocrine Tumors | 3 |
| Non-small cell lung cancer stage II | 3 |
| Non-small cell lung cancer stage III | 3 |
| Pheochromocytoma | 3 |
| Philadelphia chromosome-positive acute lymphoblastic leukemia | 3 |
| Recurrent Endometrial Cancer | 3 |
| refractory plasma cell neoplasm | 3 |
| Renal Pelvis and Ureter Urothelial Carcinoma | 3 |
| Secondary malignant neoplasm of bone | 3 |
| Secondary malignant neoplasm of pancreas | 3 |
| Serous cystadenocarcinoma ovary | 3 |
| stage I adult soft tissue sarcoma | 3 |
| stage II adult soft tissue sarcoma | 3 |
| stage III adult Burkitt's lymphoma | 3 |
| Stage IV Skin Melanoma | 3 |
| stage IV squamous cell carcinoma of the paranasal sinus and nasal cavity | 3 |
| Unknown Primary Tumors | 3 |
| untreated childhood acute myeloid leukemia and other myeloid malignancies | 3 |
| Urethral Carcinoma | 3 |
| Verrucous carcinoma of the oral cavity stage III | 3 |
| Acinar cell carcinoma of pancreas | 2 |
| acute leukemia in relapse | 2 |
| Adenocarcinoma of large intestine | 2 |
| Adenomatous polyp of colon | 2 |
| Adenomatous Polyposis Coli | 2 |
| Adrenal Gland Neoplasms | 2 |
| Adult Mixed Glioma | 2 |
| Adult T-cell lymphoma/leukaemia stage IV | 2 |
| Adult T-Cell Lymphoma/Leukemia | 2 |
| Advanced breast cancer | 2 |
| Appendiceal Neoplasms | 2 |
| B-cell small lymphocytic lymphoma stage I | 2 |
| B-cell small lymphocytic lymphoma stage II | 2 |
| Basal cell carcinoma | 2 |
| Brain Neoplasms | 2 |
| Brain Stem Neoplasms | 2 |
| Carcinoma of Male Breast | 2 |
| Carcinoma, Large Cell | 2 |
| colon cancer liver metastasis | 2 |
| Colonic Neoplasms | 2 |
| contiguous stage II adult Burkitt's lymphoma | 2 |
| Cutaneous T-cell lymphoma stage III | 2 |
| Desmoplastic Small Round Cell Tumor | 2 |
| diffuse adenocarcinoma of the stomach | 2 |
| DS Stage I Plasma Cell Myeloma | 2 |
| DS Stage II Plasma Cell Myeloma | 2 |
| DS Stage III Plasma Cell Myeloma | 2 |
| Endocrine Gland Neoplasms | 2 |
| Epithelioid mesothelioma, malignant | 2 |
| Esophageal carcinoma | 2 |
| Gallbladder Carcinoma | 2 |
| Glioblastoma multiforme of brain | 2 |
| Hairy Cell Leukemia | 2 |
| Head and neck cancer metastatic | 2 |
| Hereditary Nonpolyposis Colorectal Cancer | 2 |
| Hydatidiform mole, benign | 2 |
| insular thyroid cancer | 2 |
| insulinoma | 2 |
| intestinal adenocarcinoma of the stomach | 2 |
| Laryngeal verrucous carcinoma stage IV | 2 |
| Leukemia, Myeloid, Chronic, Atypical, BCR-ABL Negative | 2 |
| Leukemia, Myelomonocytic, Chronic | 2 |
| liposarcoma | 2 |
| Locally advanced breast cancer | 2 |
| Lymphoma | 2 |
| Malignant ascites | 2 |
| Malignant epithelial neoplasm of thyroid | 2 |
| Malignant neoplasm of gastrointestinal tract | 2 |
| malignant neoplasm of large intestine stage IIIc | 2 |
| Malignant neoplasm of skin | 2 |
| Malignant tumor of cervix | 2 |
| Malignant tumor of peritoneum | 2 |
| Mantle cell lymphoma stage IV | 2 |
| Marginal zone lymphoma stage IV | 2 |
| Medullary carcinoma of thyroid | 2 |
| Meningeal Leukemia | 2 |
| Mesothelioma malignant advanced | 2 |
| Mesothelioma malignant recurrent | 2 |
| Metastatic malignant neoplasm to brain | 2 |
| Metastatic papillary thyroid carcinoma | 2 |
| metastatic squamous neck cancer with occult primary squamous cell carcinoma | 2 |
| mixed adenocarcinoma of the stomach | 2 |
| Mucosal Melanoma | 2 |
| Mycosis fungoides/Sezary syndrome stage III | 2 |
| Mycosis fungoides/Sezary syndrome stage IV | 2 |
| MYELODYSPLASTIC SYNDROME | 2 |
| Myelofibrosis | 2 |
| Neoplasms, Hormone-Dependent | 2 |
| Neuroblastoma recurrent | 2 |
| noncontiguous stage II adult Burkitt's lymphoma | 2 |
| Oesophageal carcinoma recurrent | 2 |
| Osteosarcoma of bone | 2 |
| Pancreatic Ductal Adenocarcinoma | 2 |
| Peritoneal Neoplasms | 2 |
| Precancerous Conditions | 2 |
| Primary Effusion Lymphoma | 2 |
| Primary peritoneal carcinoma | 2 |
| Prostate carcinoma | 2 |
| Prostatic Neoplasms | 2 |
| Rectal Neoplasms | 2 |
| recurrent gastric cancer | 2 |
| Recurrent Head and Neck Carcinoma | 2 |
| Rhabdomyosarcoma | 2 |
| Salivary gland cancer stage III | 2 |
| Salivary gland cancer stage IV | 2 |
| Sarcomatoid Mesothelioma | 2 |
| Secondary malignant neoplasm of colon | 2 |
| Secondary malignant neoplasm of female breast | 2 |
| Secondary malignant neoplasm of kidney | 2 |
| Small cell lung cancer extensive stage | 2 |
| Small cell lung cancer recurrent | 2 |
| soft tissue sarcoma, adult, stage IIB | 2 |
| soft tissue sarcoma, adult, stage IIC | 2 |
| soft tissue sarcoma, adult, stage IVA | 2 |
| Squamous cell carcinoma of esophagus | 2 |
| stage I adult Burkitt's lymphoma | 2 |
| stage III follicular thyroid cancer | 2 |
| stage III papillary thyroid cancer | 2 |
| Stage IIIA Skin Melanoma | 2 |
| Stage IIIB Skin Melanoma | 2 |
| Stage IIIC Gastric Cancer | 2 |
| stage IV adult diffuse mixed cell lymphoma | 2 |
| stage IV adult diffuse small cleaved cell lymphoma | 2 |
| stage IV adult Hodgkin's lymphoma | 2 |
| stage IV adult immunoblastic large cell lymphoma | 2 |
| stage IV adult lymphoblastic lymphoma | 2 |
| stage IV follicular thyroid cancer | 2 |
| stage IV grade 1 follicular lymphoma | 2 |
| stage IV grade 2 follicular lymphoma | 2 |
| stage IV grade 3 follicular lymphoma | 2 |
| Stage IVA Oropharyngeal Squamous Cell Carcinoma | 2 |
| Strawberry nevus of skin | 2 |
| Superficial basal cell carcinoma | 2 |
| T-Cell Lymphoma | 2 |
| Transitional cell cancer of renal pelvis and ureter metastatic | 2 |
| unresectable extrahepatic bile duct cancer | 2 |
| unresectable gallbladder cancer | 2 |
| untreated metastatic squamous neck cancer with occult primary | 2 |
| Urologic Neoplasms | 2 |
| Verrucous carcinoma of the oral cavity stage IV | 2 |
| Acute Erythroblastic Leukemia | 1 |
| Acute monocytic leukemia | 1 |
| Acute myelomonocytic leukemia | 1 |
| Acute Undifferentiated Leukemia | 1 |
| adult brain tumor | 1 |
| adult central nervous system germ cell tumor | 1 |
| adult grade III lymphomatoid granulomatosis | 1 |
| adult primary hepatocellular carcinoma | 1 |
| adult solid tumor | 1 |
| Adult T-cell lymphoma/leukaemia stage III | 1 |
| Adult teratoma | 1 |
| AIDS-related peripheral/systemic lymphoma | 1 |
| AIDS with Kaposi's sarcoma | 1 |
| Anaplastic large T-cell systemic malignant lymphoma | 1 |
| Angiomyolipoma of kidney | 1 |
| Basal Cell Nevus Syndrome | 1 |
| Bladder Adenocarcinoma | 1 |
| Bladder cancer recurrent | 1 |
| Bladder cancer stage II | 1 |
| Bladder Squamous Cell Carcinoma | 1 |
| Bronchioloalveolar Adenocarcinoma | 1 |
| Cancer of Urinary Tract | 1 |
| Carcinoid tumor of lung | 1 |
| Carcinoma in situ of adrenal cortex | 1 |
| Carcinoma, Neuroendocrine | 1 |
| Carcinoma, Transitional Cell | 1 |
| Carcinomatosis of peritoneal cavity | 1 |
| Castration-resistant prostate cancer | 1 |
| cervical cancer | 1 |
| Cervical carcinoma stage IB | 1 |
| Cervical Intraepithelial Neoplasia | 1 |
| Cervix carcinoma | 1 |
| Cervix carcinoma stage III | 1 |
| childhood brain tumor | 1 |
| childhood chronic myelogenous leukemia | 1 |
| Childhood myelodysplastic syndrome | 1 |
| Chronic eosinophilic leukemia | 1 |
| Colorectal cancer recurrent | 1 |
| Cutaneous T-cell lymphoma stage I | 1 |
| Cutaneous T-cell lymphoma stage II | 1 |
| Dermatofibrosarcoma Protuberans | 1 |
| Ependymoma | 1 |
| Epithelial tumor of ovary | 1 |
| Extragonadal Seminoma | 1 |
| Fallopian Tube Carcinoma | 1 |
| Fibromatosis | 1 |
| Fibromatosis, Aggressive | 1 |
| Follicular thyroid carcinoma | 1 |
| Gastric Adenocarcinoma | 1 |
| Giant Cell Tumor of Bone | 1 |
| Glioblastoma Multiforme | 1 |
| Glucagonoma | 1 |
| Granulosa cell tumor of the ovary | 1 |
| Hemangioblastoma | 1 |
| Hemangioma | 1 |
| Hematologic Neoplasms | 1 |
| Hepatobiliary neoplasm | 1 |
| Hepatoma recurrent | 1 |
| High-Grade Squamous Intraepithelial Lesions | 1 |
| High Grade Sarcoma | 1 |
| Histiocytosis, Langerhans-Cell | 1 |
| Human epidermal growth factor 2 negative carcinoma of breast | 1 |
| Invasive Ductal Breast Carcinoma | 1 |
| Kaposi's sarcoma classical type | 1 |
| kidney/urinary cancer | 1 |
| Laryngeal Squamous Cell Carcinoma | 1 |
| Leukemia, Large Granular Lymphocytic | 1 |
| Leukemia, Mast-Cell | 1 |
| Leukoplakia, Oral | 1 |
| Liposarcoma, Dedifferentiated | 1 |
| Liver carcinoma | 1 |
| Liver cell carcinoma non-resectable | 1 |
| Lung Neoplasms | 1 |
| Lymphoma, Diffuse | 1 |
| Lymphoma, Mixed-Cell, Follicular | 1 |
| male reproductive cancer | 1 |
| Malignant Childhood Neoplasm | 1 |
| Malignant epithelial tumor of ovary | 1 |
| Malignant Extragonadal Germ Cell Tumor | 1 |
| Malignant Extragonadal Non-Seminomatous Germ Cell Tumor | 1 |
| Malignant glioma of brain | 1 |
| Malignant neoplasm of brain | 1 |
| Malignant neoplasm of esophagus | 1 |
| Malignant neoplasm of fallopian tube | 1 |
| Malignant neoplasm of liver | 1 |
| Malignant neoplasm of penis | 1 |
| Malignant neoplasm of testis | 1 |
| Malignant Peripheral Nerve Sheath Tumor | 1 |
| Malignant Pleural Mesothelioma | 1 |
| Mammary Carcinoma, Human | 1 |
| Mantle cell lymphoma stage III | 1 |
| Marginal zone lymphoma stage III | 1 |
| Meningeal Carcinomatosis | 1 |
| meningeal chronic myelogenous leukemia | 1 |
| Metastatic Malignant Neoplasm to the Leptomeninges | 1 |
| Metastatic osteosarcoma | 1 |
| Mycosis fungoides/Sezary syndrome stage I | 1 |
| Mycosis fungoides/Sezary syndrome stage II | 1 |
| Nasopharyngeal carcinoma | 1 |
| Neoplasms by Histologic Type | 1 |
| Neoplasms, Glandular and Epithelial | 1 |
| Non-Hodgkin's lymphoma of central nervous system | 1 |
| Noninfiltrating Intraductal Carcinoma | 1 |
| Nonsquamous nonsmall cell neoplasm of lung | 1 |
| Osteosarcoma recurrent | 1 |
| Ovarian germ cell cancer stage IV | 1 |
| Pancreatic carcinoma | 1 |
| Pancreatic Endocrine Carcinoma | 1 |
| Pancreatic Neoplasm | 1 |
| pancreatic polypeptide tumor | 1 |
| Papillary thyroid carcinoma | 1 |
| Plexiform Neurofibroma | 1 |
| Polycythemia Vera | 1 |
| Poorly Differentiated Thyroid Carcinoma | 1 |
| Pre B-cell acute lymphoblastic leukemia | 1 |
| Precursor T-Cell Lymphoblastic Leukemia-Lymphoma | 1 |
| Precursor T-cell lymphoblastic lymphoma | 1 |
| Primary central nervous system lymphoma | 1 |
| Primary malignant neoplasm of gastrointestinal tract | 1 |
| Primary Myelofibrosis | 1 |
| Primary Systemic Amyloidosis | 1 |
| progressive hairy cell leukemia, initial treatment | 1 |
| Pulmonary lymphangioleiomyomatosis | 1 |
| recurrent childhood acute myeloid leukemia | 1 |
| recurrent gastrointestinal carcinoid tumor | 1 |
| recurrent islet cell carcinoma | 1 |
| Recurrent respiratory papillomatosis | 1 |
| Refractory Brain Neoplasm | 1 |
| regional gastrointestinal carcinoid tumor | 1 |
| Renal cancer metastatic | 1 |
| Sarcoma | 1 |
| Secondary malignant neoplasm of stomach | 1 |
| Small Lymphocytic Lymphoma | 1 |
| Squamous cell carcinoma of lung | 1 |
| Squamous cell carcinoma of mouth | 1 |
| Squamous cell carcinoma of nose | 1 |
| Squamous cell carcinoma of pharynx | 1 |
| Squamous cell carcinoma of skin | 1 |
| Stage IA Non-Small Cell Lung Carcinoma | 1 |
| Stage IA Pancreatic Cancer | 1 |
| Stage IB Non-Small Cell Lung Carcinoma | 1 |
| Stage IB Pancreatic Cancer | 1 |
| Stage IIA Non-Small Cell Lung Carcinoma | 1 |
| Stage IIA Pancreatic Cancer | 1 |
| Stage IIB Pancreatic Cancer | 1 |
| stage III adult diffuse mixed cell lymphoma | 1 |
| stage III adult diffuse small cleaved cell lymphoma | 1 |
| stage III adult Hodgkin's lymphoma | 1 |
| stage III adult immunoblastic large cell lymphoma | 1 |
| stage III adult lymphoblastic lymphoma | 1 |
| stage III grade 1 follicular lymphoma | 1 |
| stage III grade 2 follicular lymphoma | 1 |
| stage III grade 3 follicular lymphoma | 1 |
| Stage IIIA Colorectal Cancer | 1 |
| Stage IIIB Colorectal Cancer | 1 |
| Stage IIIC Colorectal Cancer | 1 |
| Stage IV Extragonadal Non-Seminomatous Germ Cell Tumor | 1 |
| Stage IV Extragonadal Seminoma | 1 |
| Stage IVA Colorectal Cancer | 1 |
| Stage IVA Laryngeal Squamous Cell Carcinoma | 1 |
| Stage IVA Laryngeal Verrucous Carcinoma | 1 |
| Stage IVA Lip and Oral Cavity Squamous Cell Carcinoma | 1 |
| Stage IVA Nasal Cavity and Paranasal Sinus Squamous Cell Carcinoma | 1 |
| Stage IVA Oral Cavity Verrucous Carcinoma | 1 |
| Stage IVA Thyroid Gland Follicular Carcinoma | 1 |
| Stage IVA Thyroid Gland Papillary Carcinoma | 1 |
| Stage IVB Colorectal Cancer | 1 |
| Stage IVB Laryngeal Squamous Cell Carcinoma | 1 |
| Stage IVB Laryngeal Verrucous Carcinoma | 1 |
| Stage IVB Lip and Oral Cavity Squamous Cell Carcinoma | 1 |
| Stage IVB Nasal Cavity and Paranasal Sinus Squamous Cell Carcinoma | 1 |
| Stage IVB Oral Cavity Verrucous Carcinoma | 1 |
| Stage IVB Oropharyngeal Squamous Cell Carcinoma | 1 |
| Stage IVB Thyroid Gland Follicular Carcinoma | 1 |
| Stage IVB Thyroid Gland Papillary Carcinoma | 1 |
| Stage IVC Laryngeal Squamous Cell Carcinoma | 1 |
| Stage IVC Laryngeal Verrucous Carcinoma | 1 |
| Stage IVC Lip and Oral Cavity Squamous Cell Carcinoma | 1 |
| Stage IVC Nasal Cavity and Paranasal Sinus Squamous Cell Carcinoma | 1 |
| Stage IVC Oral Cavity Verrucous Carcinoma | 1 |
| Stage IVC Oropharyngeal Squamous Cell Carcinoma | 1 |
| Stage IVC Thyroid Gland Follicular Carcinoma | 1 |
| Stage IVC Thyroid Gland Papillary Carcinoma | 1 |
| Testis cancer recurrent | 1 |
| thorax/respiratory cancer | 1 |
| Thyroid Neoplasm | 1 |
| Transitional cell carcinoma of bladder | 1 |
| Treatment related secondary malignancy | 1 |
| Tuberous Sclerosis | 1 |
| Uterine Fibroids | 1 |
